# Data on antioxidant activity in grapevine (*Vitis vinifera* L.) following cryopreservation by vitrification

**DOI:** 10.1016/j.dib.2015.10.012

**Published:** 2015-10-21

**Authors:** María Fernanda Lazo-Javalera, Martín Ernesto Tiznado-Hernández, Irasema Vargas-Arispuro, Elisa Valenzuela-Soto, María del Carmen Rocha-Granados, Marcos Edel Martínez-Montero, Marisela Rivera-Domínguez

**Affiliations:** a Research Center of Food and Development, A.C. Biotechnology and Plant Molecular Biology Laboratory. Food Science Coordination, Carretera a la Victoria Km 0.6, Hermosillo, Sonora 83000, Mexico; b Research Center of Food and Development, A.C. Vegetal Origin Food Technology Coordination, Carretera a la Victoria Km 0.6, Hermosillo, Sonora 83000, Mexico; c Plant Molecular Biology Laboratory. Michoacana University de San Nicolás de Hidalgo, Paseo Gral. Lázaro Cárdenas y Berlín S/N, Col. Viveros, C.P. 60170 Uruapan, Michoacán, Mexico; d Bioplants Center, Plant Breeding Laboratory University of Ciego de Avila, Car. a Moron km 9, CP 69450 Ciego de Avila, Cuba

## Abstract

Cryopreservation is used for the long-term conservation of plant genetic resources. This technique very often induces lethal injury or tissue damage. In this study, we measured indicators of viability and cell damage following cryopreservation and vitrification-cryopreservation in *Vitis vinifera* L. axillary buds cv. “Flame seedless” stored in liquid nitrogen (LN) for: three seconds, one hour, one day, one week and one month; after LN thawed at 38 °C for three minutes. The enzymatic activity of catalase (CAT) and superoxide dismutase (SOD), as well as the amount of malondialdehyde (MDA), total protein and viability were assayed.

Specifications table

**Table:** 

Subject area	Biology
More specific subject area	Plant preservation
Type of data	Table, figure
How data was acquired	UV-visible spectrophotometer
Data format	Analyzed
Experimental factors	Cryopreservation consists in buds immersed directly into LN without cryoprotectors unlike vitrification-cryopreservation technique in which used PVS2. Both techniques were stored in LN for an hour, a day, a week, and a month. After each freezing period, cryovials containing frozen buds were thawed rapidly in a water-bath for 3 min at 38 °C.
Experimental features	Protein extract from cryopreserved buds were analyzed for SOD, CAT and MDA assays.
Data source location	Data analysis was obtained in Hermosillo, México. Plant material (axillary buds) were obtained in the “Casas Grandes” vineyard located 40 km from Highway 36 North to the coast of Hermosillo, Sonora, México (29°02׳41.0"N, 111°43׳59.3"W)
Data accessibility	Data is available with this article

**Value of the data**

• This data provides information on the effect of cryopreservation in axillary grapevine buds, and in tissue antioxidant activity. The data obtained shows the behavior of the antioxidant system cryopreservation-vitrification in different times of storage in liquid nitrogen.
• Information of the antioxidant effects in cryopreserved buds produced here provides a tool to understand how the tissues adapt to this extreme environment.
• This data can be used to evaluate different preservation techniques.

## 1 Data

The data shared in this article is the viability and tissue antioxidant activity of ‘Flame seedless’ grapevine axillary buds in several cryopreservation conditions, which were stored at different times.

### 1.1 Viability

**Fig. 1:**
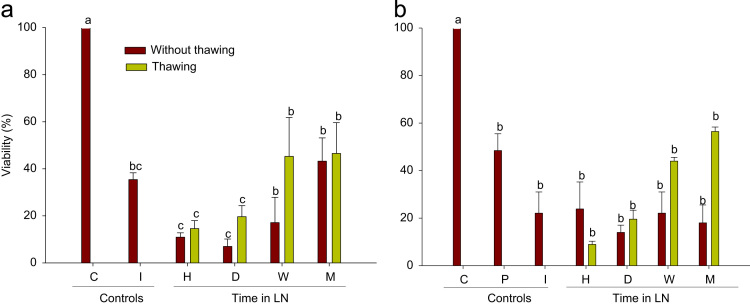
Viability (%) in ‘Flame seedless’ grapevine buds. Cryopreserved (a) and vitrified-cryopreserved (b). C: buds without treatment, I: buds stored in LN for 3 s, H: buds stored in LN for one hour, D: buds stored in LN for one day, W: buds stored in LN for one week, M: buds stored in LN for one month. Black-colored bars indicate buds without thawing; grey-colored bars indicate buds thawed at 38 °C for 3 min. Different letters indicate differences between treatments according to the Tukey–Kramer test.

Cryopreserved and vitrified-cryopreserved buds showed differences in viability with treatment. No differences (*P*>0.05) were found between cryopreserved (Fig. 1a) and vitrified-cryopreserved (Fig. 1b) treatments, but both treatments were significantly different compared to the control.

### 1.2 Antioxidant activity

**Fig. 2:**
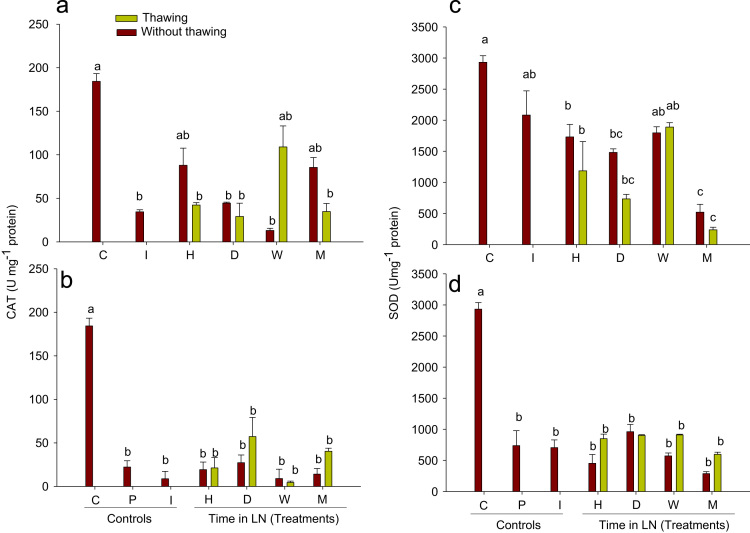
Catalase activity (a,b) and superoxide dismutase activity (c,d) (U/mg protein) in ‘Flame seedless’ grapevine buds. Cryopreserved (a,c) and vitrified-cryopreserved (b,d). C: buds without treatment, P: buds immersed in cryoprotective solution, I: buds stored in LN for 3 s, H: buds stored in LN for one hour, D: buds stored in LN for one day, W: buds stored in LN for one week, M: buds stored in LN for one month. Black-colored bars indicate buds without thawing; grey-colored bars indicate buds thawed at 38 °C for 3 min. Different letters indicate differences between treatments according to the Tukey–Kramer test.

No significant effects (*P*>0.05) were detected in the CAT activity due to the thawing step (Fig. 2b). However, the CAT activity showed a large decrease with respect to the control (Tukey, *P*<0.05). A reduction in the enzymatic activity of the vitrified-cryopreserved buds compared with the cryopreserved buds (Fig. 2a) was observed. No significant differences were observed in the SOD activity of the cryopreserved buds with or without a thawing step (Fig. 2c), and the enzymatic activity showed a tendency to decline compared to the control as the storage time increased. In contrast, the SOD activity in the vitrified-cryopreserved tissues (Fig. 2d) showed a large decrease compared with the control (Tukey, *P*<0.05). The SOD activity in the cryopreserved buds (Fig. 2c) was higher than in the vitrified-cryopreserved buds (Fig. 2d).

**Fig. 3:**
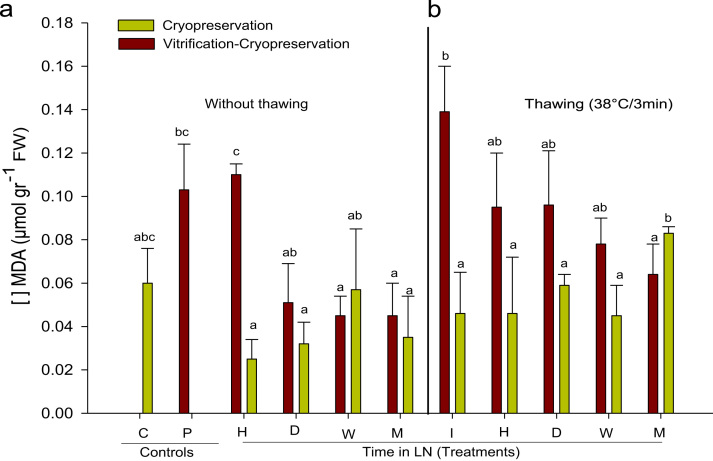
Malondialdehyde (MDA) content (µmol g^−1^ of fresh weight) in grapevine buds cryopreserved and vitrified-cryopreserved for different times of storage in LN. (a) Buds without thawing and (b) buds thawed at 38 °C for 3 min. C: buds without treatment; P: buds immersed in cryoprotective solution, I: buds stored in LN for 3 s, H: buds stored in LN for one hour, D: buds stored in LN for one day, W: buds stored in LN for one week, M: buds stored in LN for one month. Black-colored bars indicate buds treated or not (white bars) with PVS2 solution and stored in LN. Different letters indicate differences between treatments according to the Tukey–Kramer test.

No differences were found in the treatments without thawing (Fig. 3a) with respect to the controls (C) and (P). Comparison between the cryopreserved and vitrified-cryopreserved buds after 1 h in LN revealed that the highest level of MDA was observed in the vitrified-cryopreserved buds (0.11 µmol g^−^^1^FW) compared to the cryopreserved buds (0.025 µmol g^−1^FW). No significant differences were found among the treatments for the other storage times (Tukey, *P*>0.05). Significant differences were detected between the vitrified-cryopreserved and cryopreserved buds at the initial time (I) and after one month of storage in LN (Fig. 3b).

Protein content significant differences were found between the vitrification-cryopreservation treatments at the initial time with thawing and after one month in LN and for the treatments with or without the thawing step (Table 1). The highest concentration of protein (48.5 µg g^−1^ FW) was found in the treatment with no thawing step after one month of storage in liquid nitrogen. No significant differences (Tukey, *P*>0.05) were observed among the other treatments; for each storage time in LN, a higher amount of total protein was obtained in the buds that were in contact with the PVS2 solution compared with cryopreservation either with or without thawing. In addition, a higher amount of total protein was recorded in the tissues treated with vitrification-cryopreservation compared to the control (Tukey, *P*<0.05).

## 2 Experimental design, materials and methods

### 2.1 Cryopreservation procedure

The rootstocks were randomly selected and washed three times with water. Axillary buds were dissected with a sterile razor blade and disinfected in commercial chlorine solution at 25% (1.3% NaOCl) with 0.1% Tween-20 for 5 min and then rinsed three times with sterile distilled water. The samples were then treated with the systemic fungicide benomyl (100 ppm) for 3 min, washed again with sterile distilled water and with 70% ethanol (v/v), and then washed with sterile distilled water three times. For cryopreservation, the disinfected buds (five replicates per treatment with five buds each, *n*=25) were transferred to sterile 2-mL polypropylene cryovials and immersed directly into LN. The sampling was carried out at the initial time of freezing (3 s) and after storage for an hour, a day, a week, and a month in LN. After each freezing period, cryovials containing frozen buds were obtained without thawing and then thawed rapidly in a water-bath at 38 °C for 3 min.

### 2.2 Vitrification-cryopreservation procedure

For vitrification-cryopreservation, the disinfected buds were treated according to the procedure described by Matsumoto and Sakai [1] with some modifications. The plant vitrification solution N°2 (PVS2) contained 30% (w/v) glycerol, 15% (w/v) ethylene-glycol, and 15% (w/v) dimethyl-sulfoxide in MS medium with 0.4 M sucrose at pH 5.8 [2]. The buds were disinfected as described above and transferred into 2-mL cryovials (five replicas per treatment with five buds each, *n*=25) containing 1 mL of PVS2 solution previously sterilized by filtration. The control treatment did not include the PVS2 solution. The samples were incubated at 25±2 °C with agitation for 180 min. This was the best incubation time according to a previous viability assay using grapevine buds (data not shown). The control treatment samples were not frozen. The cryovials were directly immersed in LN, and an initial sample was taken immediately after 3 s of freezing; the remaining buds were stored for an hour, a day, a week, and a month. After each treatment, one sample was obtained without thawing and another was thawed in a water-bath at 38 °C for 3 min. The PVS2 solution was removed, and the buds were washed with sterile distilled water.

### 2.3 Viability assay

Viability was estimated using a triphenyltetrazolium chloride (TTC) reduction assay [3]. Five buds were incubated in 5 mL of 0.1% TTC solution in 0.05 M potassium phosphate buffer (pH 7.5). The reaction was performed at 30 °C for 24 h, after which the buds were washed with sterile distilled water. Formazan was extracted from viable cells with 5 mL of 95% ethanol at 80 °C for 10 min in the dark. The supernatant was separated by centrifugation (10,000 rpm for 5 min) [4]. The absorbance of the extracted formazan was measured at 530 nm in a Cary 50 UV-visible spectrophotometer. The tissue viability was expressed as the percentage reduction in TTC activity compared to that of control cells (non-cryopreserved fresh tissue) and was calculated as described by Alotto et al. [5] using the following formula:

Viabilityindex(VI)=Opticaldensity(595nm)/GramsoftissuePercentviability(%viability)=(VIofTreatedbuds/VIofcontrolfreshsamples)×100

### 2.4 Enzymatic activity determinations

SOD (EC 1.15.1.1) activity was quantified following the method reported by Beyer and Fridovich [6], [7] by quantifying the photochemical reduction of nitro blue tetrazolium by the change in absorbance at 550 nm using a Cary 50 UV-visible spectrophotometer. CAT (EC 1.11.1.6) activity was determined at 25 °C according to the method described by Aebi [8] by monitoring the decrease in absorbance of hydrogen peroxide (H_2_O_2_) at 240 nm during 1 min at 25 °C using a Cary 50 UV-Visible spectrophotometer from Varian.

### 2.5 Malondialdehyde concentration

Lipid peroxidation was determined by quantifying the concentration of malondialdehyde. For the extraction of MDA from the treated buds and controls, the buds were ground with liquid nitrogen to a fine powder, which was then homogenized with 50 mM phosphate buffer (pH 8.3) containing 2.8% NaH_2_PO_4_·H_2_O and 1.8% of Na_2_HPO_4_ and centrifuged at 12,000 rpm for 50 min. After centrifugation, the supernatant was used for the quantification of MDA [9], [10] using an "Oxitek TBARS assay" kit, which contains the reactive substance thiobarbituric acid. The entire procedure was carried out by carefully following the manufacturer׳s instructions (ZeptoMetrix Corporation). The MDA level was expressed as µmol per gram of fresh weight (FW).

### 2.6 Total protein content

The amount of total protein was determined according to the Bradford method [11]. Bovine serum albumin from Sigma (USA) was used as the standard, and the enzymatic activities were expressed as specific activity by determining the protein concentration in each sample.

### 2.7 Data analysis

The data obtained were analyzed using an analysis of variance (ANOVA). Significant differences among the treatments were determined by the Tukey–Kramer multiple range test at a significance level of 95%. All data were analyzed using the statistical package NCSS (Statistical Number System) version 2007.

## Figures and Tables

**Table 1:** Effect of thawing on total protein content (µg g^−1^) of fresh weight in ‘Flame seedless’ grapevine buds treated for different times with vitrification or vitrification-cryopreservation.

	Vitrification-cryopreservation		Cryopreservation	
Time in LN	Without thawing Mean±SD (µg g^−1^FW)	Thawing (38 °C/3 min) Mean±SD (µg g^−1^FW)	Without thawing Mean±SD (µg g^−1^FW)	Thawing (38 °C/3 min) Mean±SD (µg g^−1^FW)
Control	1.9±1.7	1.9±1.7	1.9±1.7	1.9±1.7
PVS2	20.9±6.7	–	–	–
Initial	–	33.9±3.9⁎	–	4.1±2.2
1 h	27.9±1.6	10.8±4.1	5.3±3.2	10.4±1.5
1 day	16.4±5.1	19.9±4.5	8.8±2.4	5.2±4.4
1 week	28.1±11.2	21.8±5.5	1.6±0.6	1.5±1.5
1 month	48.5±10.4⁎	37.0±5.9⁎	24.4±3.7	18.3±5.4

PVS2 refers to treatment by immersion into cryoprotective solution only. Values represent means ± standard deviation.

* Indicates differences (*P*<0.05) among treatments according to the Tukey–Kramer test.
