# Supplementary material for: Data on antioxidant activity in grapevine (Vitis vinifera L.) following cryopreservation by vitrification
Source: Data Brief. 2015 Oct 21;5:549–55. doi: 10.1016/j.dib.2015.10.012 (PMC4773358; doi:10.1016/j.dib.2015.10.012)
Supplement: Supplementary file 1 — Supplementary material [file mmc1.pdf]

CENTRO DE INVESTIGACION EN
ALIMENTACION Y DESARROLLO

CIAD

SEP-CONACYT-UNAM-IPN-GOBIERNO DE SONORA-GOBIERNO DE SINALOA-SEMARNAP

Hermosillo Sonora, México

Sept 28, 2015

Sarah O'Loughlin
Managing Editor

Dear Editor,

The authors whose names are listed immediately below certify that they have NO affiliations with or involvement in any organization or entity with any financial or personal interest in the subject matter or materials discussed in this manuscript.

Authors mames:

Marisela Rivera-Domínguez

María Fernanda Lazo-Javalera

Martín Ernesto Tiznado-Hernández

Irasema Vargas-Arispuro

Elisa Valenzuela-Soto

María del Carmen Rocha-Granados

Marcos Edel Martínez-Montero

Km. 0.6 Carretera a la Victoria
Apartado Postal 1735
Hermosillo, Sonora,83000 Méx.

Tel. (62) 89-24-00
Fax (62) 80-00-55
